# Management of epilepsy in pregnancy in eastern China: A survey from the Zhejiang association against epilepsy

**DOI:** 10.3389/fneur.2022.1001918

**Published:** 2022-11-17

**Authors:** Zheng-Yan-Ran Xu, Ping Qian, Meng-Ting Cai, Mei-ping Ding, Yi Guo

**Affiliations:** ^1^Department of Neurology, Epilepsy Center, The Second Affiliated Hospital of Zhejiang University School of Medicine, Hangzhou, China; ^2^Nursing Department, The Second Affiliated Hospital of Zhejiang University School of Medicine, Hangzhou, China; ^3^Department of General Practice and International Medicine, The Second Affiliated Hospital of Zhejiang University School of Medicine, Hangzhou, China; ^4^Key Laboratory of Medical Molecular Imaging of Zhejiang Province, Hangzhou, China

**Keywords:** epilepsy, pregnancy, management, questionnaire, survey

## Abstract

**Objective:**

We aimed to evaluate the knowledge of the board members of the Zhejiang Association Against Epilepsy (ZAAE) regarding pregnancy of women with epilepsy (WWE), as well as their clinical practice and obstacles in the management of WWE.

**Methods:**

A cross-sectional survey was conducted among the board members of the ZAAE using a questionnaire based on the management guidelines for WWE during pregnancy in China. We recorded the demographic characteristics of the surveyed practitioners, the coincidence rate of each question, clinical practice, and the barriers encountered in managing WWE.

**Results:**

This survey showed that the average knowledge score of the surveyed practitioners was 71.02%, and the knowledge score of neurologists was higher than that of neurosurgeons. Knowledge regarding the following three aspects was relatively poor: whether WWE is associated with an increased risk of cesarean section and preterm delivery, the preferred analgesic drugs for WWE during delivery, and the time of postpartum blood concentration monitoring. After multiple linear regression analysis, the score of neurologists was correlated to the number of pregnant WWE treated each year. In addition, the biggest difficulty in the management of WWE during pregnancy is the lack of patient education and doctors training on pregnant epilepsy management.

**Conclusion:**

Our study revealed the ZAAE board members' knowledge and management status of pregnant WWE. In addition, our study identified the biggest obstacle to the management of WWE during pregnancy, and emphasized the importance of training and practice of epilepsy knowledge during pregnancy for practitioners and the significance of interdisciplinary communication.

## Introduction

Epilepsy is one of the most common chronic diseases affecting women of childbearing age (1), with ~0.2 to 0.5% of all pregnant women having a history of epilepsy (2). Most pregnant women with epilepsy (WWE) could have healthy offspring, however, complications that impact maternal and fetal health can occur (3). For example, the risk of teratogenesis caused by anti-seizure medications (ASMs), exacerbation of seizures during pregnancy, and pregnancy related depression (4–6). Although WWE are at risk during pregnancy, previous studies have shown that WWE have unsatisfactory understanding of pregnancy-related epilepsy problems, and health care providers lack comprehensive knowledge of the management of WWE during pregnancy (7–9).

Awareness and adequate counseling of management issues in WWE are key components of patient care. After the release of the UK guidelines for the management of WWE (10), a study suggested that pre-pregnancy counseling might reduce the frequency of adverse outcomes, such as major congenital malformations (10–14). Metcalfe et al. (15) found that patients did not know enough about the new guidelines for pregnancy of WWE established by the American Academy of Neurology. Currently, guidelines for the management of pregnant WWE have been published in China (16); however, the situation regarding practitioners' management of WWE during pregnancy is not clear. Therefore, the aim of the present study was to evaluate practitioners' management of WWE during pregnancy. This information is essential to ensure the highest quality of care for WWE, because it identifies deficiencies in practitioner knowledge that must be addressed.

## Methods

### Survey

This is a cross-sectional study of the board members of the Zhejiang Association Against Epilepsy (ZAAE), a branch of the China Association Against Epilepsy (CAAE). These board members represent the doctors who mainly treat epilepsy in the whole Zhejiang Province. The ZAAE has 76 board members, including a retired consultant, a neuroscientist, and three electroencephalogram (EEG) technicians, all five of whom who were not included in the survey. Seventy-one board members were recruited in the study. Board members were emailed with an invitation and link to the electronic questionnaire. From April 1 to April 7, 2022, we alerted the participants via WeChat or telephone.

Participants were eligible if they were: a professional who were currently engaged in clinical work related to epilepsy; and provided informed consent. The study was approved by the Second Affiliated hospital of Zhejiang University School of Medicine Ethics Committee.

### Questionnaire

A questionnaire comprising 41 items was constructed by two epileptologists (Mei-Ping Ding and Yi Guo), based on the management guidelines for WWE during pregnancy in China (16), modified on the basis of Nathalie Jetté's and Makiko Egawa's questionnaires (7, 17). This questionnaire emphasized knowledge of pregnancy-related risks associated with the use of specific ASMs. Two types of questions were used: single-choice and multiple-choice questions. The questionnaire included 20 questions about practitioners' understanding of the guidelines, and 21 questions related to the demographic characteristics of the practitioners and the practice and barriers in the clinical management of WWE. The questionnaire was designed to take no more than 10–15 min to complete.

### Statistical analysis

Descriptive statistics were used to analyze all variables. The knowledge score was expressed as the percentage of answers that met guidelines' recommendations among the 20 questions in **Table 2**, thus excluding demographic issues as well as practices and barriers in the clinical management of WWE during pregnancy. The calculation method was to set a weight of 1 to each answer that met guideline's recommendations in single-choice questions and multiple-choice questions respectively, and divided the number of answers that met guideline's recommendations given by the practitioners by the total number of answers that met guideline's recommendations in the questionnaire.

We used the knowledge score as a continuous variable to analyze the linear relationship between demographic characteristics and knowledge score. After the multiple sample Shapiro-Wilk test, the scores were normally distributed, so the parametric test was used. Firstly, we analyzed the variables one-by-one using an independent sample *T*-test and one-way analysis of variance (ANOVA). Then, we entered all variables that were significantly associated with the outcome (*P* < 0.05) into a multiple linear regression analysis; a Chi-squared test was used to analyze the significant differences in demographic characteristics between neurologists and neurosurgeons, as well as each question whose cumulative coincidence rate was <70%. The Supplementary Table 7 containing information on how variables are coded and the presentation of sociodemographic variables are provided as Supplementary material. A *p*-value of < 0.05 was considered statistically significant. IBM SPSS Statistics program version 25 was used for the analyses (IBM Corp., Armonk, NY, USA).

## Results

A total of 62 out of 71 practitioners finally completed the survey, with a response rate of 87.32%.

### Practitioners' demographics

As shown in Table 1, 53.23% (*n* = 33) of the surveyed practitioners were female, and most of the surveyed practitioners worked in the university health care system (*n* = 29; 46.78%) and public hospitals (*n* = 32; 51.61%). Among the practitioners, 33.87% (*n* = 21) had a doctorate and 30.65% (*n* = 19) had a master's degree. Neurologists accounted for 82.26% (*n* = 51) of the practitioners. More than half of the practitioners (*n* = 33; 53.22%) had more than 10 years of experience in treating epilepsy. Most practitioners (*n* = 41; 66.13%) treated <30% of patients with epilepsy. Moreover, the majority of the practitioners (*n* = 48; 77.42%) treated fewer than ten cases of pregnant WWE every year.

**Table 1:** Demographic characteristics of surveyed practitioners.

**Demographic characteristics**	**Surveyed practitioners *N* (%)**
**Gender**	
Male	29 (46.77)
Female	33 (53.23)
**Primary setting of employment**	
University health care system	29 (46.78)
Public hospital	32 (51.61)
Private hospital	1 (1.61)
**Level of experience managing epilepsy**	
Expert (>10 years)	33 (53.22)
Advanced (5–10 years)	23 (37.10)
Novice (<5 years)	6 (9.68)
**Specialty**	
Neurologist	51 (82.26)
Neurosurgeon	8 (12.90)
Pediatrician	3 (4.84)
**Education background**	
Doctor	21 (33.87)
Master	19 (30.65)
Bachelor	22 (35.48)
**Percentage of patients who have epilepsy**	
<10%	21 (33.87)
≥10 and <30%	20 (32.26)
≥30 and <50%	7 (11.29)
≥50 and <70%	10 (16.13)
≥70 and <90%	4 (6.45)
**Number of pregnant WWE treated each year**	
<10 cases	48 (77.42)
≥10 cases	14 (22.58)

WWE, women with epilepsy.

### Practitioners' knowledge of pregnancy management of WWE

#### Management during pre-pregnancy and pregnancy

The average knowledge score of the surveyed practitioners was 71.02%. As displayed in Table 2, over half of the practitioners knew the duration of folic acid supplementation in WWE (*n* = 36; 58.06%) and the dose of folic acid supplementation (*n* = 43; 69.35%). Compared with the 48.39% (*n* = 30) of the practitioners who believed that there was a high likelihood of maintaining seizure free status during pregnancy if WWE were seizure free prior to conception for 6 months, only 45.16% (*n* = 28) of the practitioners realized that was 9 months. The majority of the practitioners (*n* = 46; 74.19%) answered that it was necessary to avoid the use of multiple ASMs during pregnancy. Most practitioners knew that levetiracetam (*n* = 47; 75.81%) and lamotrigine (*n* = 44; 70.97%) had sufficient evidence of high safety, whereas only 43.55% (*n* = 27) knew that oxcarbazepine also showed high safety. Among the practitioners, 59.68% (*n* = 37) knew that the blood concentration of lamotrigine decreased most significantly during pregnancy compared with that before pregnancy. For pregnant WWE taking lamotrigine, only 43.55% (*n* = 27) of the practitioners suggested blood concentration monitoring within 1 month. About half of the practitioners (*n* = 33; 53.22%) knew that there was good evidence that valproate (VPA) could cause cognitive decline in the offspring. Nearly half of the practitioners (*n* = 27; 43.54%) believed that women who were using VPA and had an unplanned pregnancy needed to replace valproic acid with other ASMs to reduce the risk of teratogenesis, even if their seizures were well-controlled. Most (*n* = 53; 85.48%) practitioners chose that if the seizure was poorly controlled, they needed to replace VPA with newer ASMs that work faster, or add new ASMs and maintain a relatively low dose VPA.

**Table 2:** Practitioners' knowledge of pregnancy management in WWE.

**Questions**	**Practitioners responding *N* (%)**
1. **From the beginning of pregnancy preparation, WWE need to supplement with folic acid every day, and at least until when?**	
6 weeks of pregnancy	3 (4.84)
**12 weeks of pregnancy**	36 (58.06)
18 weeks of pregnancy	2 (3.23)
24 weeks of pregnancy	7 (11.29)
After delivery	14 (22.58)
**2. What is the recommended daily dose of folic acid for WWE during pregnancy if they are taking folic acid antagonists, have a history of abortion, or have given birth to neural tube teratoma?**	
0.4 mg	12 (19.35)
0.6 mg	6 (9.68)
**5 mg**	43 (69.36)
I don't know	1 (1.61)
3. There is good evidence to support the fact that WWE have a high likelihood (84–92%) of remaining seizure free during pregnancy if they were seizure free prior to conception for:	
6 months	30 (48.39)
**9 months**	28 (45.16)
I don't know	4 (6.45)
4. To reduce the risk of severe congenital malformations, is it considered necessary to avoid the use of multiple ASM therapy during pregnancy?	
**Yes**	46 (74.19)
No	6 (9.68)
Insufficient evidence or no evidence	9 (14.52)
I don't know	1 (1.61)
**5. For which of the following ASMs, is there sufficient evidence that they show high safety, and the incidence of congenital malformations in their offspring is similar to that of WWE who do not take ASMs^a^:**	
**Levetiracetam**	47 (75.81)
**lamotrigine**	44 (70.97)
**Oxcarbazepine**	27 (43.55)
Topiramate	4 (6.45)
Carbamazepine	4 (6.45)
Insufficient evidence or no evidence	4 (6.45)
I don't know	1 (1.61)
**6. During pregnancy in WWE, which kind of ASM has the most significant decrease in blood concentration compared with that before pregnancy?**	
**lamotrigine**	37 (59.68)
Levetiracetam	5 (8.06)
Oxcarbazepine	3 (4.84)
Topiramate	1 (1.61)
Phenobarbital	2 (3.23)
Phenytoin	2 (3.23)
Zonisamide	1 (1.61)
I don't know	11 (17.74)
**7. For pregnant WWE taking lamotrigine, how frequently do you generally recommend that the patients' blood concentration is monitored?**	
**1 month**	27 (43.55)
2 months	11 (17.74)
3 months	15 (24.19)
I don't know	1 (1.61)
Never recommend	8 (12.91)
**8. There is good evidence that the cognitive ability of children of WWE exposed to what kind of ASM decreases?**	
**Valproic acid**	33 (53.22)
Topiramate	14 (22.58)
Phenobarbital	7 (11.29)
Phenytoin	2 (3.23)
Carbamazepine	1 (1.61)
Insufficient evidence or no evidence of the above ASMs	2 (3.23)
I don't know	3 (4.84)
**9. When a WWE with an unplanned pregnancy, she's taking valproic acid and her seizures are well-controlled, would you recommend to replace valproic acid with other ASMs to reduce the risk of teratogenesis?**	
Yes	27 (43.54)
**No**	31 (50.00)
Insufficient evidence or no evidence	2 (3.23)
I don't know	2 (3.23)
**10. For a WWE with an unplanned pregnancy, she's using valproic acid, if her seizure control is poor, either replace valproic acid with newer ASMs that work faster, or add a new ASM and maintain a low dose of valproic acid, will you recommend one of the two options?**	
**Yes**	53 (85.48)
No	7 (11.29)
I don't know	2 (3.23)
**11. Is there a significant increase in the risk of cesarean section or early delivery in WWE?**	
Yes	28 (45.16)
**No**	15 (24.19)
Insufficient evidence or no evidence	15 (24.19)
I don't know	4 (6.46)
**12. For pregnant women with frequent seizures and high risk of status epilepticus, can selective cesarean section be considered?**	
**Yes**	60 (96.77)
No	2 (3.23)
**13. Do WWE have an increased risk of having a small for-gestational-age infant?**	
**Yes**	40 (64.52)
No	4 (6.45)
Insufficient evidence or no evidence	14 (22.58)
I don't know	4 (6.45)
**14. Is there an increased risk of perinatal death in neonates born to WWE?**	
**Yes**	31 (50.00)
No	9 (14.52)
Insufficient evidence or no evidence	15 (24.19)
I don't know	7 (11.29)
**15. Which of the following analgesic drugs should be given priority in the delivery of WWE?**	
**Morphine**	24 (38.71)
Pethidine	4 (6.45)
Insufficient evidence or no evidence	11 (17.74)
I don't know	23 (37.10)
**16. If WWE have GCSE during delivery, which drug is the first choice to terminate the seizure as soon as possible?**	
**Benzodiazepines**	49 (79.03)
Phenobarbital	1 (1.61)
Propofol	10 (16.13)
Valproic acid	2 (3.23)
**17. Should WWE continue to take ASMs during delivery?**	
**Yes**	59 (95.16)
No	2 (3.23)
Insufficient evidence or no evidence	1 (1.61)
**18. With the gradual normalization of drug metabolism after delivery, the risk of a high blood drug concentration increases. To further adjust the dose of ASMs, how frequently do you generally recommend monitoring the blood drug concentration after delivery?**	
1 week	23 (37.10)
**2 weeks**	23 (37.10)
1 month	11 (17.74)
I don't know	5 (8.06)
**19. For WWE who used enzyme-induced ASMs during pregnancy, does intramuscular vitamin K at birth reduce the risk of neonatal bleeding?**	
**Yes**	52 (83.87)
No	2 (3.23)
Insufficient evidence or no evidence	4 (6.45)
I don't know	4 (6.45)
**20. Should breastfeeding be encouraged in WWE treated with ASMs monotherapy?**	
**Yes**	55 (88.71)
No	4 (6.45)
Insufficient evidence or no evidence	2 (3.23)
I don't know	1 (1.61)

WWE, women with epilepsy; GCSE, generalized convulsions status epilepticus; ASM, Anti-Seizure Medication; Boldface indicates the answer consistent with guidelines.

a Responses were not mutually exclusive, and participants could select more than one.

#### Management during childbirth

As shown in Table 2, only a few practitioners (*n* = 15; 24.19%) understood that WWE did not have a significantly increased risk of cesarean section or early delivery. Almost all practitioners (*n* = 60; 96.77%) comprehended that selective cesarean section could be considered for WWE with frequent seizures and high risk of epileptic status, and 64.52% (*n* = 40) knew that WWE had an increased risk of having a small for-gestational-age infant. Only 50% (*n* = 31) believed that there was an increased risk of perinatal death in neonates born to WWE. Less than half of the practitioners (*n* = 24; 38.71%) knew that morphine was the preferred analgesic drug during childbirth of WWE. Among the practitioners, 79.03% (*n* = 49) knew that benzodiazepines were the first choice to terminate the seizure as soon as possible when WWE had generalized convulsions status epilepticus (GCSE) during delivery, and 95.16% (*n* = 59) knew that they should continue to take ASMs during delivery.

#### Management after childbirth

As described in Table 2, only 37.10% (*n* = 23) of the practitioners knew that it was recommended to monitor the blood drug concentration for 2 weeks after delivery. Most practitioners (*n* = 52; 83.87%) knew that for WWE who used enzyme-induced ASMs during pregnancy, intramuscular vitamin K at birth would reduce the risk of neonatal bleeding, and 88.71% (*n* = 55) practitioners recommended that breastfeeding should be encouraged for WWE treated with ASM monotherapy.

### Management practices and barriers in epilepsy during pregnancy

As shown in Table 3, most practitioners (*n* = 57; 91.94%) believed that it was necessary to join pregnancy registration for WWE to manage their pregnancy. Among the practitioners, 98.39% (*n* = 61) would pay attention to the neuropsychological problems of WWE during pregnancy and measure the blood drug concentration before pregnancy. The most commonly measured blood drug concentrations of ASMs were carbamazepine (*n* = 61; 98.39%) and VPA (*n* = 59; 95.16%). Among the practitioners, the same percentage, 40.32% (*n* = 25), suggested monitoring the blood drug concentration once every 1 to 2 months and once every 2 to 3 months, respectively. The majority of practitioners (*n* = 46; 74.19%) would provide information on pregnancy-related problems when requested by patients. The most information provided was related to the risk of ASMs (*n* = 59; 95.16%), followed by precautions during pregnancy (*n* = 54; 87.1%) and folic acid supplementation (*n* = 53; 85.48%). When WWE received information about pregnancy-related problems, 80.64% (*n* = 50) of practitioners thought that their attitude toward pregnancy had changed. Although 88.71% (*n* = 55) of practitioners considered it necessary to communicate with an obstetrician before WWE became pregnant, only 20.97% (*n* = 13) currently cooperated with obstetricians before conception. Communicating with obstetricians through medical records was the choice of 75.81% (*n* = 47) of the practitioners, and most of them wanted to receive information about the mode of delivery from the obstetricians. At present, practitioners believe that the biggest difficulty in the management of WWE during pregnancy is the lack of patient education related to epilepsy during pregnancy (*n* = 50; 80.65%), followed by the lack of training for the management of epilepsy during pregnancy (*n* = 48; 77.42%).

**Table 3:** Management practices and barriers in epilepsy during pregnancy.

**Questions**	**Practitioners responding *N* (%)**
**1. Is it necessary to join in pregnancy registration for WWE to manage pregnancy?**	
Necessary	57 (91.94)
Necessary but not feasible	5 (8.06)
Unnecessary	0 (0.00)
**2. Do you pay attention to the neuropsychological problems of WWE during pregnancy?**	
Yes, I do	61 (98.39)
No, I don't	1 (1.61)
**3. If you can measure the blood drug concentration of ASMs, will you measure the blood drug concentration before pregnancy as the baseline value?**	
Yes, I will	61 (98.39)
No, I won't	1 (1.61)
**4. The blood concentration of which of the following ASMs can you measure in your clinic? (including delivery tests) ^a^**	
lamotrigine	36 (58.06)
Levetiracetam	30 (48.39)
Oxcarbazepine	34 (54.84)
Topiramate	18 (29.03)
Phenobarbital	29 (46.77)
Phenytoin	34 (54.84)
Carbamazepine	61 (98.39)
Valproic acid	59 (95.16)
Perampanel	6 (9.68)
I don't know	1 (1.61)
**5. For pregnant WWE, how often do you generally recommend to monitor the blood drug concentration?**	
Once every 1 to 2 months	25 (40.32)
Once every 2 to 3 months	25 (40.32)
1 to 3 times throughout pregnancy	4 (6.46)
The practitioner depends on the condition	8 (12.90)
**6. Do you provide information on pregnancy-related problems for WWE?** ^**a**^	
Yes, on a regular basis	20 (32.26)
Yes, upon the patient's request	46 (74.19)
Yes, upon request from the patient's parents	35 (56.45)
Yes, upon request from an obstetrician	27 (43.55)
Not provided	1 (1.61)
Other	3 (4.84)
**7. When do you provide such information to WWE?** ^**a**^	
Junior high school	5 (8.06)
Senior high school	6 (9.68)
University	16 (25.81)
Once the patient has a boyfriend	35 (56.45)
Upon marriage	36 (58.06)
Upon request	44 (70.97)
Upon advice of the parents	34 (54.84)
At the first visit	23 (37.10)
On becoming pregnant	30 (48.39)
Other	5 (8.06)
**8. What is included in such information?** ^**a**^	
Risks of ASMs	59 (95.16)
Folic acid supplementation	53 (85.48)
Precautions during pregnancy	54 (87.10)
Mode of delivery	36 (58.06)
ASMs and breastfeeding	47 (75.81)
Child rearing	33 (53.23)
Contraception	32 (51.61)
Inheritance of epilepsy	44 (70.97)
Other	10 (16.13)
**9. Do you think the attitude of patients toward pregnancy changes after receiving such information?**	
Yes	50 (80.64)
No	6 (9.68)
Other	6 (9.68)
**10. Is it necessary to have prior communication with obstetricians before your patients' pregnancy?**	
Yes	55 (88.71)
No	5 (8.06)
Other	2 (3.23)
**11. What is the current status of cooperation with obstetricians?** ^**a**^	
Contact before WWE become pregnant	13 (20.97)
Contact when WWE become pregnant	19 (30.65)
There are no obstetricians to cooperate with	19 (30.65)
Contact upon request by WWE	41 (66.13)
Other	2 (3.23)
**12. How do you communicate with your obstetrician when your patient is pregnant?** ^**a**^	
Write email	2 (3.23)
Call obstetrician	35 (56.45)
Never communicate	3 (4.84)
Write it on the medical record	47 (75.81)
Other	4 (6.45)
**13. What kind of information do you anticipate from obstetricians after patients' delivery?** ^**a**^	
Weeks of delivery	45 (72.58)
Mode of delivery	56 (90.32)
Birth weight	46 (74.19)
Apgar score	49 (79.03)
Other	8 (12.90)
**14. What do you think are the biggest difficulties in pregnancy management of WWE at present? (Select the top 3 options)** ^**a**^	
Patients have high mobility and poor compliance	39 (62.90)
Unplanned pregnancy	37 (59.68)
Unable to monitor drug blood concentration	22 (35.48)
Inadequate communication with obstetrics	39 (62.90)
Patients lack relevant popular science education on epilepsy during pregnancy	50 (80.65)
Doctors lack training in epilepsy management during pregnancy	48 (77.42)
Failure to obtain the best drugs (such as lamotrigine, levetiracetam, or oxcarbazepine)	15 (24.19)

WWE, women with epilepsy; ASM, Anti-Seizure Medication.

a Responses were not mutually exclusive, and participants could select more than one.

### Variables related to practitioners' knowledge

To determine whether the demographic characteristics of the practitioners affected the knowledge score, we further conducted difference analysis and regression analysis. The knowledge scores were statistically significant among four aspects: Gender (*P* = 0.014), levels of experience in treating epilepsy (*P* = 0.045), the number of P pregnant WWE treated each year (*P* = 0.008), and specialty (*P* < 0.001) (data were shown in Supplementary material). After incorporating the above four variables into the multiple linear regression model, we found that specialty (*p* = 0.002; 9.45, 95% CI 3.77 to 15.12) (Table 4) was a relevant factor of practitioners' knowledge, with statistical significance between neurologists and neurosurgeons (*P* < 0.001; 73.83 ± 11.23% vs. 58.19 ± 10.33%; 15.64%, 95% CI 7.29 to 24.00%) and between neurologists and pediatricians (*P* = 0.015, 73.83 ± 11.23% vs. 57.47 ± 5.27%; 16.36%, 95% CI 3.31 to 29.41%) (Table 5). In addition, we also conducted difference analysis and regression analysis on neurologists alone. We added the level of treatment experience (*P* = 0.002) and the number of pregnant WWE treated annually (*P* = 0.013) to the multiple linear regression. As displayed in Table 6, we found that the number of pregnant WWE treated annually was a relevant factor of neurologists ' knowledge (*P* = 0.015; 5.49, 95% CI 1.10 to 9.88).

**Table 4:** Independent correlates of practitioners' knowledge after multivariate linear regression analysis.

	**Coefficients^a^**							
	**Model**	**Unstandardized coefficients**		**Standardized coefficients**	***t***	**Sig**.	**95.0% confidence interval for B**	
		**B**	**Std. Error**	**Beta**			**Lower bound**	**Upper bound**
1	(Constant)	37.146	8.222		4.518	0.000	20.681	53.611
	Level of experience managing epilepsy	−3.829	2.150	−0.206	−1.781	0.080	−8.134	0.475
	Specialty	9.445	2.836	0.400	3.331	**0.002**	3.767	15.124
	Gender	4.210	2.961	0.171	1.422	0.160	−1.719	10.139
	Number of pregnant WWE treated each year	5.886	3.360	0.200	1.752	0.085	−0.842	12.613

WWE, women with epilepsy.

95% CI: 95% confidence interval.

A p-value of < 0.05 was considered significant and is marked in bold.

a Dependent Variable: Coincidence Rate.

**Table 5:** Multiple comparisons of independent correlates of practitioners' knowledge.

**(I) Specialty**	**(J) Specialty**	**Mean difference (I–J)**	**Std. error**	**Sig**.	**95% confidence interval**	
					**Lower bound**	**Upper bound**
Neurosurgeon	Pediatrist	0.71839%	7.43154%	0.923	−14.1521%	15.5889%
Neurologist	Pediatrist	16.36241%	6.52138%	**0.015**	3.3132%	29.4117%
Neurologist	Neurosurgeon	15.64402%	4.17431%	**0.000**	7.2913%	23.9968%

A p-value of < 0.05 was considered significant and is marked in bold.

Dependent Variable: Coincidence Rate.

**Table 6:** Independent correlates of neurologists ' knowledge after multivariate linear regression analysis.

	**Coefficients^a^**							
	**Model**	**Unstandardized coefficients**		**Standardized coefficients**	**t**	**Sig**.	**95.0% confidence interval for B**	
		**B**	**Std. error**	**Beta**			**Lower bound**	**Upper bound**
1	Constant	65.593	7.194		9.118	0.000	51.129	80.057
	Level of experience managing epilepsy	−1.364	2.454	−0.084	−0.556	0.581	−6.297	3.569
	Number of pregnant WWE treated each year	5.486	2.183	0.382	2.513	**0.015**	1.096	9.875

WWE, women with epilepsy.

95% CI: 95% confidence interval.

A p-value of < 0.05 was considered significant and is marked in bold.

a Dependent Variable: Coincidence Rate.

## Discussion

To our knowledge, this was the first survey to evaluate the pregnancy management of WWE among practitioners in China. We found that the average knowledge score of the board members of ZAAE was only 71.02%, and the issue with the lowest coincidence rate was whether the risk of cesarean section or early delivery is increased in WWE. The biggest obstacle to the management of WWE during pregnancy was that patients lack relevant education on epilepsy during pregnancy and doctors lack training in epilepsy management during pregnancy. Multiple linear regression analysis showed the knowledge score of neurologists was statistically higher than that of neurosurgeons and pediatricians, respectively. Furthermore, the neurologist's knowledge score correlated significantly with the number of pregnant WWE treated each year.

The average knowledge score of the board members of ZAAE was 71.02% in our study, which was higher than the survey of from Jetté's group (49.3%) (7). This discrepancy might be partly caused by the type of respondents included in each study, the study by Jetté's group included neurologists and neurology residents, whereas most of the respondents in our study are professionals with clinical experience of epilepsy (90.32%). Another reason may be differences in the content of the questionnaires. Less than 70% of practitioners knew the duration and the dosage of folic acid supplementation in WWE, indicating a lack of knowledge on the rational use of folic acid in WWE. Actually, there is no real current evidence on the best dose of folic acid in case of previous abortions or folic acid antagonists in women with epilepsy. In the Chinese guidelines (16), a daily dose of 5 mg of folic acid is recommended as grade D based on experts opinions. Only 45.16% of practitioners knew that there was a high likelihood to remain seizure free during pregnancy if WWE were 9 months seizure-free before pregnancy, which was consistent with the survey by Jetté's group (20%) (7). On the contrary, 48.39% of practitioners chose 6 months without seizure before pregnancy, which was recommended by the consensus of Chinese experts in 2015 (18), this indicated that practitioners need to learn the new guidelines. In addition, practitioners had a relatively poor understanding of the issues related to childbirth in WWE. For example, less than half (38.71%) of the practitioners pointed out that morphine was preferred for analgesia during childbirth of WWE. Moreover, only a few practitioners (24.19%) knew that WWE did not have a significantly increased risk of cesarean section or early delivery, which was similar to the survey by Jetté's group (28.9%) (7). The above two conditions might be related to poor communication between epilepsy practitioners and obstetricians.

For medication-related issues, most practitioners pointed out that lamotrigine was considered a safer option and were aware of the change in plasma drug concentration during pregnancy. Nevertheless, less than half of the practitioners understood that oxcarbazepine also shows high safety, which might reflect the lower amount of evidence for oxcarbazepine compared with lamotrigine and levetiracetam. Most (83.87%) practitioners suggested that for WWE who used enzyme-induced ASM during pregnancy, intramuscular vitamin K at birth would reduce the risk of neonatal bleeding, which was similar to results described by Babiker et al. (74%) (19). About half (53.23%) of the practitioners knew that there was good evidence that VPA would reduce the offspring's cognitive ability, which was inconsistent with the study by Jetté's group (33.3%) (7). The discrepancy with our results could be related to different respondents, that more epilepsy professionals were enrolled in our study. Nearly half (43.55%) of practitioners believed that VPA needed to be replaced during pregnancy if their seizures were well-controlled, which was inconsistent with guideline's recommendation. Actually, it is not recommended to replace VPA temporarily during pregnancy in Chinese guidelines (a grade D recommendation). These findings suggested that practitioners did not accurately understand the risks of VPA to the offspring and did not balance the effects of VPA adjustment on seizures. Only 43.55% of practitioners knew that the recommended interval for blood concentration monitoring of lamotrigine after pregnancy was every month. 1 month was recommended in the latest Chinese guidelines (16), therefore, it is necessary for practitioners to update the knowledges. In addition, the frequency of monitoring blood concentrations is also associated with a lack of medical conditions, which may reflect difficulties in the implement.

In terms of management practice of pregnant WWE, we found that only a small number of respondents (32.26%) would regularly provide information on pregnancy for WWE, compared with most respondents (74.19%) who provided information at the request of patients. This might reflect inadequate doctor-patient communication in the clinic in China. Zelano's group designed an online tool for information to WWE, which enhanced patient education and improved communication during pregnancy for WWE (20). Our study also found that most WWE changed their attitude toward pregnancy after receiving pregnancy-related information. Therefore, we recommend that doctors regularly provide pregnancy-related information to WWE in clinic or online. Besides, although most doctors (88.71%) believed that it was necessary to communicate with obstetricians before pregnancy, only 20.97% currently cooperated with them, which might be explained by the lack of referrals in China's medical system. Almost all respondents wanted to know about the delivery mode of WWE from obstetricians, which further illustrated the necessity for communication (21).

The lack of patient education on epilepsy during pregnancy and the lack of training on pregnant epilepsy management by doctors were the biggest difficulties in pregnancy management of WWE. Borgelt et al. (22) found that a specialty pharmacist in the ambulatory care neurology team may enhance patient education efficacy, which emphasized the importance of multidisciplinary collaboration. An online course on epilepsy for primary care physicians in Latin America was shown to be a cost-effective course, with good retention and excellent approval rates (23). This suggests that we can develop an online course for Chinese practitioners to manage pregnant WWE.

After multiple linear regression analysis, it was found that neurologists' knowledge of WWE during pregnancy was better than that of neurosurgeons. These findings suggested that neurologists might pay more attention to the management of pregnant WWE, particularly the teratogenic effects of ASMs on offspring, which might be related to the fact that neurologists pay more attentions to pregnant WWE annually than neurosurgeons. Five out eight neurosurgeons (more than 63%) still treated WWE during pregnancy. Therefore, it is important to train neurosurgeons in the knowledge of epilepsy during pregnancy. There were differences between the neurologist's final score and the number of pregnant WWE treated each year, suggesting the importance of training and practice of epilepsy during pregnancy.

Limitations of this study were as follows: First, the respondents of this study were the members of the board of the ZAAE, representing Zhejiang Province, East of China, which cannot represent the other areas of China. Second, obstetricians, psychiatrists, and geneticists were not enrolled in our study. In fact, the management of pregnant WWE requires multidisciplinary participation (21, 24). Third, psychological counseling for pregnancy was not included in this study. As a matter of fact, peripartum depression and anxiety are the most common complications of pregnancy, which have a significant adverse impact on pregnancy outcome and child development (4). In the future, it is necessary to conduct a multi-regional and multi-disciplinary survey of WWE management in pregnancy, including pregnancy psychological issues.

## Conclusion

Our study revealed the ZAAE board members' knowledge and management status of pregnant WWE. The knowledge in the following three aspects was relatively poor: whether the risk of cesarean section and preterm delivery in WWE was increased, the preferred analgesic drugs for WWE during delivery, and the time of postpartum blood concentration monitoring. In addition, our study also identified the biggest obstacle to the management of WWE during pregnancy. The results emphasized the importance of training and practice of epilepsy knowledge during pregnancy for practitioners and the significance of interdisciplinary communication.

## Data availability statement

The original contributions presented in the study are included in the article/Supplementary material, further inquiries can be directed to the corresponding author/s.

## Ethics statement

The studies involving human participants were reviewed and approved by the Second Affiliated Hospital of Zhejiang University School of Medicine Ethics Committee. The patients/participants provided their written informed consent to participate in this study.

## Author contributions

YG designed and analyzed the data. YG and Z-Y-RX drafted and edited the manuscript. YG, M-TC, PQ, and M-pD contributed to revising the manuscript. All authors contributed to the article and approved the submitted version.

## Funding

This work was supported by grants from the National Natural Science Foundation of China [Grant number 81871010].

## Conflict of interest

Author YG was the board member of ZAAE. M-pD was the president of ZAAE. This survey was supported by ZAAE. The remaining authors declare that the research was conducted in the absence of any commercial or financial relationships that could be construed as a potential conflict of interest.

## Publisher's note

All claims expressed in this article are solely those of the authors and do not necessarily represent those of their affiliated organizations, or those of the publisher, the editors and the reviewers. Any product that may be evaluated in this article, or claim that may be made by its manufacturer, is not guaranteed or endorsed by the publisher.
