# Supplementary material for: Management of epilepsy in pregnancy in eastern China: A survey from the Zhejiang association against epilepsy
Source: Front Neurol. 2022 Nov 17;13:1001918. doi: 10.3389/fneur.2022.1001918 (PMC9714667; doi:10.3389/fneur.2022.1001918)
Supplement: Supplementary file 1 [file Data_Sheet_1.docx]

**Supplementary information**

1. **Questionnaire for management of epilepsy in pregnancy**

Q1: Gender (please select one)

1. Male
2. Female

Q2: Primary setting of employment (please select one)

1. University Health Care System
2. Public Hospital
3. Private Hospital

Q3: Level of experience managing epilepsy (please select one)

1. Expert (>10 years)
2. Advanced (5–10 years)
3. Novice (< 5 years)

Q4: Specialty (please select one)

1. Neurologist
2. Neurosurgeon
3. Pediatrician

Q5: Education background (please select one)

1. Doctor
2. Master
3. Bachelor

Q6: Percentage of patients who have epilepsy (please select one)

1. < 10%
2. ≥ 10% and < 30%
3. ≥ 30% and < 50%
4. ≥ 50% and < 70%
5. ≥ 70% and < 90%

Q7: Number of pregnant WWE treated each year (please select one)

1. < 10 cases
2. ≥ 10 cases

Q8: From the beginning of pregnancy preparation, WWE need to supplement with folic acid every day, and at least until when? (please select one)

1. 6 weeks of pregnancy
2. 12 weeks of pregnancy
3. 18 weeks of pregnancy
4. 24 weeks of pregnancy
5. After delivery
6. I don't know

Q9: What is the recommended daily dose of folic acid for WWE during pregnancy if they are taking folic acid antagonists, have a history of abortion, or have given birth to neural tube teratoma? (please select one)

1. 0.4 mg
2. 0.6 mg
3. 5 mg
4. I don't know

Q10: There is good evidence to support the fact that WWE have a high likelihood (84–92%) of remaining seizure free during pregnancy if they were seizure free prior to conception for: (please select one)

1. Three months
2. Six months
3. Nine months
4. I don't know

Q11: To reduce the risk of severe congenital malformations, is it considered necessary to avoid the use of ASM multidrug therapy during pregnancy? (please select one)

1. Yes
2. No
3. Insufficient evidence or no evidence
4. I don't know

Q12: For which of the following ASMs is there sufficient evidence that they show high safety, and the incidence of congenital malformations in their offspring is similar to that of WWE who do not take ASMs ^a^: (select all that apply)

1. Levetiracetam
2. Lamotrigine
3. Oxcarbazepine
4. Topiramate
5. Carbamazepine
6. Insufficient evidence or no evidence
7. I don't know

Q13: During pregnancy in WWE, which kind of ASM has the most significant decrease in blood concentration compared with that before pregnancy? (please select one)

1. lamotrigine
2. Levetiracetam
3. Oxcarbazepine
4. Topiramate
5. Phenobarbital
6. Phenytoin
7. Zonisamide
8. Insufficient evidence or no evidence
9. I don't know

Q14: For pregnant WWE taking lamotrigine, how frequently do you generally recommend that the patients' blood concentration is monitored? (please select one)

1. One month
2. Two months
3. Three months
4. I don't know
5. Never recommend

Q15: There is good evidence that the cognitive ability of children of WWE exposed to what kind of ASM decreases? (please select one)

1. Valproic acid
2. Topiramate
3. Phenobarbital
4. Phenytoin
5. Carbamazepine
6. Insufficient evidence or no evidence of the above ASMs
7. I don't know

Q16: When a WWE with an unplanned pregnancy, she's taking valproic acid and her seizures are well controlled, would you recommend to replace valproic acid with other ASMs to reduce the risk of teratogenesis? (please select one)

1. Yes
2. No
3. Insufficient evidence or no evidence
4. I don't know

Q17: For a WWE with an unplanned pregnancy, she’s using valproic acid, if her seizure control is poor, either replace valproic acid with newer ASMs that work faster, or add a new ASM and maintain a low dose of valproic acid, will you recommend one of the two options? (please select one)

1. Yes
2. No
3. Insufficient evidence or no evidence
4. I don’t know

Q18: Is there a significant increase in the risk of cesarean section or early delivery in WWE? (please select one)

1. Yes
2. No
3. Insufficient evidence or no evidence
4. I don't know

Q19: For pregnant women with frequent seizures and high risk of status epilepticus, can selective cesarean section be considered? (please select one)

1. Yes
2. No
3. Insufficient evidence or no evidence
4. I don't know

Q20: Do WWE have an increased risk of having a small for-gestational-age infant? (please select one)

1. Yes
2. No
3. Insufficient evidence or no evidence
4. I don't know

Q21: Is there an increased risk of perinatal death in neonates born to WWE? (please select one)

1. Yes
2. No
3. Insufficient evidence or no evidence
4. I don't know

Q22: Which of the following analgesic drugs should be given priority in the delivery of WWE? (please select one)

1. Morphine
2. Pethidine
3. Insufficient evidence or no evidence
4. I don't know

Q23: If WWE have GCSE during delivery, which drug is the first choice to terminate the seizure as soon as possible? (please select one)

1. Benzodiazepines
2. Phenobarbital
3. Propofol
4. Valproic acid

Q24: Should WWE continue to take ASMs during delivery? (please select one)

1. Yes
2. No
3. Insufficient evidence or no evidence
4. I don't know

Q25: With the gradual normalization of drug metabolism after delivery, the risk of a high blood drug concentration increases. To further adjust the dose of ASMs, how frequently do you generally recommend monitoring the blood drug concentration after delivery? (please select one)

1. One week
2. Two weeks
3. One month
4. I don't know

Q26: For WWE who used enzyme-induced ASMs during pregnancy, does intramuscular vitamin K at birth reduce the risk of neonatal bleeding? (please select one)

1. Yes
2. No
3. Insufficient evidence or no evidence
4. I don't know

Q27: Should breastfeeding be encouraged in WWE treated with ASMs monotherapy? (please select one)

1. Yes
2. No
3. Insufficient evidence or no evidence
4. I don't know

Q28: Is it necessary to join in pregnancy registration for WWE to manage pregnancy? (please select one)

1. Necessary
2. Necessary but not feasible
3. Unnecessary

Q29: Do you pay attention to the neuropsychological problems of WWE during pregnancy? (please select one)

1. Yes, I do
2. No, I don't

Q30: If you can measure the blood drug concentration of ASMs, will you measure the blood drug concentration before pregnancy as the baseline value? (please select one)

1. Yes, I will
2. No, I won't

Q31: The blood concentration of which of the following ASMs can you measure in your clinic? (including delivery tests) (select all that apply)

1. lamotrigine
2. Levetiracetam
3. Oxcarbazepine
4. Topiramate
5. Phenobarbital
6. Phenytoin
7. Carbamazepine
8. Valproic acid
9. Perampanel
10. I don't know

Q32: For pregnant WWE, how often do you generally recommend to monitor the blood drug concentration? (please select one)

1. Once every 1 to 2 months
2. Once every 2 to 3 months
3. 1 to 3 times throughout pregnancy
4. The practitioner depends on the condition

Q33: Do you provide information on pregnancy-related problems for WWE? (select all that apply)

1. Yes, on a regular basis
2. Yes, upon the patient’s request
3. Yes, upon request from the patient’s parents
4. Yes, upon request from an obstetrician
5. Not provided
6. Other

Q34: When do you provide such information to WWE? (select all that apply)

1. Junior high school
2. Senior high school
3. University
4. Once the patient has a boyfriend
5. Upon marriage
6. Upon request
7. Upon advice of the parents
8. At the first visit
9. On becoming pregnant
10. Other

Q35: What is included in such information? (select all that apply)

1. Risks of ASMs
2. Folic acid supplementation
3. Precautions during pregnancy
4. Mode of delivery
5. ASMs and breastfeeding
6. Child rearing
7. Contraception
8. Inheritance of epilepsy
9. Other

Q36: Do you think the attitude of patients toward pregnancy changes after receiving such information? (please select one)

1. Yes
2. No
3. Other

Q37: Is it necessary to have prior communication with obstetricians before your patients’ pregnancy? (please select one)

1. Yes
2. No
3. Other

Q38: What is the current status of cooperation with obstetricians? (select all that apply)

1. Contact before WWE become pregnant
2. Contact when WWE become pregnant
3. There are no obstetricians to cooperate with
4. Contact upon request by WWE
5. Other

Q39: How do you communicate with your obstetrician when your patient is pregnant? (select all that apply)

1. Write email
2. Call obstetrician
3. Never communicate
4. Write it on the medical record
5. Other

Q40: What kind of information do you anticipate from obstetricians after patients’ delivery? (select all that apply)

1. Weeks of delivery
2. Mode of delivery
3. Birth weight
4. Apgar score
5. Other

Q41: What do you think are the biggest difficulties in pregnancy management of WWE at present? (Select the top 3 options)

1. Patients have high mobility and poor compliance
2. Unplanned pregnancy
3. Unable to monitor drug blood concentration
4. Inadequate communication with obstetrics
5. Patients lack relevant popular science education on epilepsy during pregnancy
6. Doctors lack training in epilepsy management during pregnancy
7. Failure to obtain the best drugs (such as lamotrigine, levetiracetam, or oxcarbazepine)

| Variables | encoding |
| --- | --- |
| Gender |  |
| Male | 1 |
| Female | 2 |
| Primary setting of employment |  |
| University Health Care System | 1 |
| Public Hospital | 2 |
| Private Hospital | 3 |
| Level of experience managing epilepsy |  |
| Expert (>10 years) | 1 |
| Advanced (5–10 years) | 2 |
| Novice (< 5 years) | 3 |
| Specialty |  |
| Neurologist | 3 |
| Neurosurgeon | 2 |
| Pediatrician | 1 |
| [Education](javascript:;) [background](javascript:;) |  |
| Doctor | 1 |
| Master | 2 |
| Bachelor | 3 |
| Percentage of patients who have epilepsy |  |
| < 10% | 1 |
| ≥ 10% and < 30% | 2 |
| ≥ 30% and < 50% | 3 |
| ≥ 50% and < 70% | 4 |
| ≥ 70% and < 90% | 5 |
| Number of pregnant WWE treated each year |  |
| ≤ 10 cases | 1 |
| > 10 cases | 2 |

**Table 7. How variables were coded and the presentation of sociodemographic variables.**
